# Effectiveness of Protective Measures and Rules in Reducing the Incidence of Injuries in Combat Sports: A Scoping Review

**DOI:** 10.3390/jfmk8040150

**Published:** 2023-10-30

**Authors:** Rastislav Štyriak, Radovan Hadža, Rafael Arriaza, Dušana Augustovičová, Erika Zemková

**Affiliations:** 1 Faculty of Physical Education and Sport, Comenius University in Bratislava, 81469 Bratislava, Slovakia; radovan.hadza@uniba.sk (R.H.); dusana.augustovicova@uniba.sk (D.A.); 2 Physical Education and Sports School, Universidade da Coruna, 15179 A Coruna, Spain; rafael@arriaza.es

**Keywords:** protectors, restrictions, guards, martial arts

## Abstract

In recent years, much effort has been made to reduce the injury rate in combat sports. However, the question remains to what extent these measures are effective. Analysis of studies could provide evidence of the effectiveness of measures aimed at reducing the incidence of injuries. This scoping review aims (1) to map research articles dealing with the effectiveness of proper use of protective measures in reducing the incidence of injuries in combat sports; (2) to investigate to what extent the proper use of protective measures and compliance with sports rules help to reduce the incidence of injuries in combat sports; and (3) to identify gaps in the existing literature and suggest future research on this topic. The literature search was conducted on articles published between 2010 and 2023. The main inclusion criteria were studies investigating the effect of sports rules and protective equipment used during training or competition in combat sports on reducing the incidence of injuries. After meeting the criteria, only seven articles from boxing, karate, and taekwondo were included in this study. Results showed that mandatory head guards, along with modern thicker gloves, significantly reduced the rate of knockouts (KOs) and head injuries in boxing despite an increase in the number of head blows. However, the number of facial cuts increased significantly due to the removal of head guards as a piece of mandatory equipment. The use of mouth guards has significantly reduced the number of oral injuries. Changes in rules, such as shortening the duration of bouts, along with the introduction of greater competencies of referees in the supervision of bouts (the standing count, outclassed rule, and medical stops) have helped to reduce the rate of injuries in boxing. The application of a computerised scoring system also contributed to the rate of injuries reduction. The increase in the number of weight divisions had the same effect on U21 karate athletes. However, a higher incentive to attack the head area in taekwondo did not increase the incidence of injuries in youth athletes. It can be concluded that the proper use of protective measures (head and mouth guards) in combination with the rules established for health protection (higher competencies of referees and more weight divisions in competitions) effectively reduces the occurrence of injuries in combat sports. In all Olympic combat sports, the injury incidence should be regularly assessed using the Injury Incidence Rate (IIR) per 1000 min exposures (MEs). Nonetheless, further studies are needed to monitor how established rules (e.g., prohibited behaviour) affect the IIRs.

## 1. Introduction

In 2022, combat sports were very popular: twenty percent of the gold medals disputed in the Olympic Games came from combat sports. Furthermore, 21.3 percent of the total number of medals distributed came from these modalities, and some of them have two bronze medal winners (e.g., karate) [1].

A combat sport, or “fighting sport”, has a competitive character that usually involves one-on-one combat with many direct contacts between competitors. Striking combat sports focus on distance-based combat, using the hands, legs, or head to land shots, and these sports include boxing, karate, and taekwondo. Grappling sports, such as judo and wrestling, primarily involve close-quarter combat techniques that emphasize throws, holds, and submissions. Depending on the sport, a contestant might win the match by scoring more points than the opponent, submitting the opponent with a hold, disabling the opponent (knockout), or attacking the opponent with a specific or designated technique. Different forms of combat sports have different rules and regulations for the obligatory equipment [2]. For example, in boxing, fighters are required to wear gloves of the required thickness, a groin guard, and a mouthguard for protection. These protectors serve the purpose of reducing the risk of injury to athletes. However, Olympic combat sport athletes sustained 40.9 competition injuries per 1000 exposures or 7.8 injuries per 1000 MEs (469.6 injuries per 1000 h of exposure), which equates to 1 injury every 24 exposures or 2.1 h of exposure, respectively [3]. The highest incidence of injuries during the Olympic Games 2020 in Tokyo was evaluated for boxing (27.1 injuries (95% CI 21.1 to 33.1)) per 100 athletes. Besides BMX racing (27.1 (12.4 to 41.8)), BMX freestyle (22.2 (0.4 to 44.0)), and skateboarding (21.0 (11.0 to 31.0)), karate appears to be one of the Olympic sports with highest IIRs (18.5 (9.1 to 27.9)) [4]. Therefore, it is necessary to reduce their occurrence. One effective way to reduce the occurrence of injuries in combat sports is to implement strict rules and regulations regarding proper training techniques and equipment usage.

As sports injuries are frequently difficult to treat and recovery can be economically demanding, injuries also place a strain on the healthcare system [5]. The epidemiological studies of Olympic combat sports such as karate [6,7,8,9,10,11,12,13,14], judo [15,16,17,18], taekwondo [19,20,21,22], boxing [23,24], wrestling [25], and fencing [26,27,28,29,30] are the main subjects of studies on injuries that concentrate on the type, severity, location, and mechanism of injuries and the risk of injury either during competitions [6,7,8,11,12,15,31,32,33] or training [34,35,36]. The most commonly injured body region in karate was the head and neck (median: 57.9%; range: 33.3% to 96.8%), while contusion (median: 68.3%; range: 54.9% to 95.1%) and laceration (median: 18.6%; range: 0.0% to 29.3%) were the most frequently reported types of injury [6]. In taekwondo, about one-third of all injuries (29.6%) in men were to the head and neck region, while almost half of the injuries (44.5%) were to the lower extremities [37]. Injuries to the head and the upper limbs occur most frequently in boxing [38]. Sprains, strains, and contusions, usually of the knee, shoulder, and fingers, were the most frequently reported injuries in judo [17]. The most commonly injured areas in wrestling were the shoulder (24%) and knee (17%) [39].

In the reviewed literature, the linkage of findings, as well as a clear definition of the relationship between the incidence of injuries and the use of mandatory protectors or applied rules, is missing. It is, therefore, necessary to investigate whether and to what extent protective gear and regulations reduce the frequency of injuries in combat sports.

## 2. Materials and Methods

This paper addresses two research questions: Do the protective measures contribute to the reduction of incidence of injuries in combat sports? How do the changes in rules affect the incidence of injuries in combat sports? 

### 2.1. Data Sources

According to the suggestions of Gusenbauer et al. [40] and Gusenbauer [41], three electronic databases (PubMed, Web of Science, and Scopus) were chosen for the search of relevant studies. Google Scholar and backward search (i.e., assessing the reference lists of the included articles) were used for additional searches. The terms “combat sports”, “martial arts”, “protective measures”, “rules”, or “protectors (gloves, helmets, body protector, mouthguard” were combined with “injuries”, “injury incidence rate”, and “risk of injury”, to find a title, an abstract, or keywords related to our topic (Supplementary Table S1).

### 2.2. Eligibility Criteria

The main inclusion criterium of our review was articles examining the impact of protective measures such as rules and protective equipment (such as gloves, body protectors, and mouth guards) used during training or competition in Olympic combat sports on the incidence of injury.

Further criteria were a publication date between 2010 and 2023 and full-text article availability. Studies that failed to meet our criteria were excluded from this review. 

In the first stage, we excluded studies with the wrong population, wrong methods, biomechanics, biochemical, laboratory, sociological, psychological studies, and training intervention studies. In the second stage, we excluded studies that investigated the effect of the rule changes on scoring or match outcomes, epidemiological studies without the association to rule changes or used protectors, prevention awareness studies, and post-injury recovery protocols. In the last stage, we excluded studies that did not address the research target. 

### 2.3. Search Strategy

All citations were imported into the citation manager Mendeley. Duplicate citations were removed manually. 

For the first screening level, only the titles of the citations were reviewed. We excluded studies with the wrong population, wrong methods, biomechanics, biochemical, laboratory, sociological, psychological, surgical practices, and training intervention studies. 

In the second phase of the screening, title and abstract relevance were independently screened by three reviewers. Reviewers met throughout the screening process to resolve conflicts and discuss any uncertainties related to study selection [42].

We excluded studies that investigated the effect of the rule changes on scoring or match outcomes and epidemiological studies that do not deal with the effect of rule changes or protectors on the occurrence of injuries, prevention awareness studies, and post-injury recovery protocols. 

In the last stage, all citations deemed relevant after the title and abstract screening were procured for subsequent review of the full-text article. All eligible articles had full texts. The characteristics of each full-text article were extracted by three independent reviewers (R. Š., D. A., and R. H.). Studies were excluded at this phase if they were found not to meet the eligibility criteria. Upon independent review, the reviewers met to resolve any conflicts and to help ensure consistency between the reviewers and the research question and purpose [42].

This process helped minimize bias and increased the reliability of the final results.

**Figure 1:**
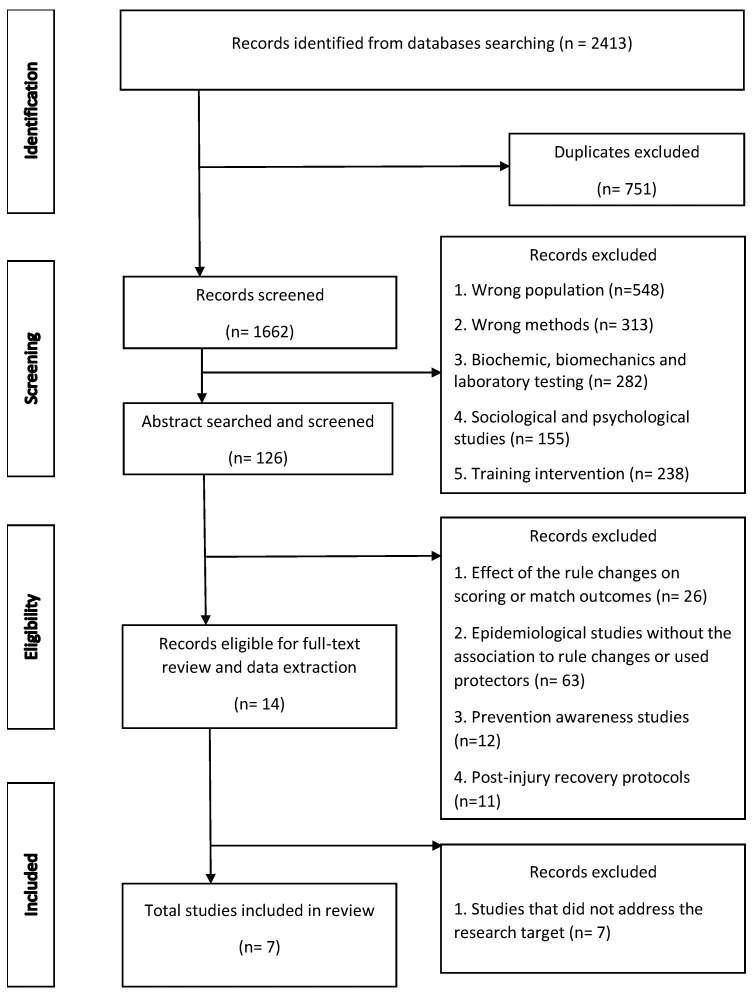
Search methodology.

The data were compiled in a single spreadsheet and imported into Microsoft Excel 2017 for validation. The Prisma flow diagram adapted from Page et al. [43] was strictly followed, as were all search and selection stages illustrated below (Figure 1).

### 2.4. Quality Assessment

The methodological quality of the studies was evaluated using appraisal assessment tool for cross-sectional studies (AXIS tool) [44]. In the AXIS tool, its items are arranged to align with the standard sections of a study report, including introduction, methods, results, discussion, and “other”. Using 20 items, the quality of the study was assessed; 6 of which have key importance, and two have secondary importance to the specific subject matter. Additionally, there are twelve more questions to appraise these important items completely. The key and secondary items are used to assign firm definitions of “high”, “medium”, or “low” quality studies. Using the AXIS tool, 7 out of 7 studies related to the effectiveness of protective measures and rules in reducing the incidence of injuries in combat sports were assessed.

## 3. Results 

The original search conducted in February 2023 yielded 2413 potentially relevant citations. After deduplication and relevance screening, 14 citations met the eligibility criteria based on title and abstract, and the corresponding full-text articles were procured for review. After data characterization of the full-text articles, seven studies remained and were included in the analysis (Table 1). Many citations were excluded upon screening at the title and abstract level as several terms used in the search algorithm also corresponded to other study topics or sports such as military, soldier injuries [45], or injuries in other non-Olympic combat sports [46] or other sports [47]. Epidemiological studies aimed exclusively at identifying the prevalence and assessing the severity, location, and type of injuries were excluded, too [10].

Only a small percentage of the epidemiological studies concentrate on the manner (or situation) in which the injury occurred [18] and how the protectors or the sport’s rules [9] reduce the likelihood of injuries in combat sports. Articles focused on protective measures mainly deal with the damping capabilities of the material, the method of measuring the effectiveness of force absorption [47], or the validation of the testing machine [48]. 

**Table 1:** An overview of studies dealing with the effects of protective measurements on the reduction of incidence of injuries in combat sports.

Authors	Study Design	Participants	Objective	Main Findings
Bianco et al., 2013 [49]	Longitudinal study	Amateur boxers participating in boxing tournaments for 59 years were reviewed.	Assessment of the evolution of rules in modern Olympic boxing and its influence on the prevalence of one result over another	- significant reduction of bouts ended due to medical decision (injuries) after introduction of mandatory head guard rule - no changes in results of medical interest with the come back to the old 3 × 3 bout formula length (2009)
Boostani et al., 2011 [50]	Cross-sectional cohort study.	40 elite karatekas at a national level	Investigation of incidence, type, and mechanism of sports injuries among Iranian elite Karatekas	- 82% of participants had an injury during last year - 32.7% of head and neck injuries - muscular injuries and contusion are the most frequent types of injury. - 27% resp. 22% of injuries occurred due to training over-exertion and inappropriate warm-up. - 57% occurred during the training. - 66.7% of injuries were caused by kicks, punch blows, and falling on the ground
Cierna et al., 2018 [12]	Cross-sectional cohort study.	2812 karatekas in four consecutive World Karate Championships	Determination of the incidence of injuries in top-level karate competitions for athletes aged 16 to 20 years.	- higher injury rate in U21 categories when competition was run with three weight categories (IIR 61.4; 95% CI 50.7 to 73.7 per 1000 AEs) than with five (IIR 18.8; 95% CI 10.9 to 30.0 per 1000 AEs), with a RR for AEs of 0.3 (95% CI 0.2 to 0.5) and RR for MEs 0.3 (95% CI 0.2 to 0.4).
Davis et al., 2018 [51]	Post-fight video analysis.	99 males elite amateur boxers	Impact of revoking permitted head guards and applying thicker gloves in 2013 on boxers’ safety	- increased ratio of head-to-body punches from 5:1 to 8:1 - increased movement around the ring by 20% - more defensive movements instead of absorbing punches
Horri et al., 2016 [52]	Cross-sectional descriptive/analytical study.	352 adolescents 11–18 years old	Determination of the prevalence of sport-related orofacial injuries in combat sports athletes wearing and not wearing mouthguards.	- 58% higher rate of trauma to the oral structures was detected in combat sports athletes who did not wear a mouthguard
Koh 2020 [53]	Post-match, interview-based prospective cohort study.	145 sparring taekwondo athletes (12–16 years of age)	Evaluating the incidence of head kicks and concussions after introducing incentive rule concussions in sparring taekwondo.	- the new competition rule relating to head kicks did not appear to increase the incidence rates of head kicks or concussions in our research participants.
Loosemore et al., 2017 [54]	Cross-sectional observational study.	AIBA boxers in total of 28,802 rounds	Incidence of stoppages due to blows to the head from AIBA bouts with and without head guards over the same period.	- significantly decreased number of stoppages due to head blows without head guards - notable increase in cuts

## 4. Discussion

### 4.1. The Role of Protectors in Reduction of Incidence Rate of Injuries in Combat Sports 

Analysis of the literature revealed that protective measures contribute to the reduction of the incidence of injuries in combat sports. 

Research proved that 82% of participants in karate practice and competition had an injury during the season because of not using protective measures. One-third of them were related to the head and neck area, while the most frequent types were muscular injuries and contusions [50].

Regarding mouthguards, laboratory tests of materials [55] or questionnaires [56] are mainly used to determine which of them are preferred by athletes. There is general agreement that the mouthguard reduces the risk of orofacial injury or concussion [52,57,58]. Although 89.8% of respondents know that wearing a mouthguard can lower the risk of oral injury, 87% of respondents agree that only 42% of combat sports athletes use a mouthguard during training [59]. Therefore, a high rate (58%) of trauma to the oral structures was found in combat sports athletes who did not wear a mouth guard [51]. 

As for the helmets, their material has been tested under laboratory conditions [48,60]. For example, O’Sullivan et al. [61] found significant differences in impact resistance between the headguards brands. The effectiveness of head guards has been investigated in boxing [49,51,54] and taekwondo [53], as they are not used during competition in other combat sports. Helmets were introduced in boxing in 1984. However, there is no evidence to demonstrate their effect on boxer safety or ability to reduce injury, and there is no regulation regarding their ability to dampen the forces of a spin punch [51]. On the contrary, the introduction of helmets led to an increase in stoppages due to head impacts, while the incidence of cuts increased significantly after revoking the rule of mandatory helmets [54]. The data indicate a 43% lower risk of stoppages (RR = 0.57) and a 430% higher risk of cuts (RR = 5.30) when boxing without the head guard. However, it is not clear whether removing the head protectors would have any effect on reducing such head injuries. For these reasons and to meet the expectations of the media and spectators (a head guard makes all boxers quite similar and anonymous), a head guard has not been mandatory in international amateur boxing since 2013 [49].

As for gloves and other body protectors used in combat sports, their effectiveness has also been tested in a laboratory [62,63,64]. The introduction of face masks for the under-sixteen age group has reduced the incidence of facial injuries with limited contact with the mask [10]. Also, additional protectors (shin, ankle, and foot) and thicker gloves reduced the risk of injury [10].

During our literature search, we also found studies dealing with injury prevention guidelines and prevention awareness [65].

### 4.2. The Role of the Rules in Reduction of Incidence Rate of Injuries in Combat Sports

Rule changes affect the incidence of injuries in combat sports.

The association between the rule changes and the risk of injuries was investigated in karate [50], boxing [49,51,54], and taekwondo [53]. 

Regarding weight categories, increasing their number in competition can reduce the rate of injuries in karate Cierna et al. [12]. Raising the number of weight categories from three to five reduced injury incidence in the U21 category. The findings of this study can be useful for epidemiologists, medical staff, coaches, athletes, and referees to better understand the rules and regulations of these sports.

As for the duration of the match, at the beginning of the last century, each boxing match could last dozens of 3 min rounds (until the bout was abandoned or the KO of one competitor). The length of the bout of 3 rounds of 3 min was changed to 5 × 2 min, later to 4 × 2 min (until 2009), decreasing KOs, RSs, and before the time limit-ended matches significantly. However, this rule of the 3 × 3 min match formula returned in 2009, probably due to the expected higher attractiveness of the sport [49].

The introduction of the referee stop contest rule (RSC) gave referees the right to interrupt the match, guarding the safety of boxers. Rules, such as the standing count rule, established in 1964, allowing boxers in difficulty to take 8 s to recover after excessive punches, showed a minimum reduction in KO rate. On the contrary, a significant increase in the rate of RSC due to injury verdict was observed (*p* < 0.03). Another modification, namely referee stop contest outclassed (RSCO) implemented in 2000, made boxing bouts less attractive (more than 15% of bouts were ended by RSCO), and this rule was revoked in 2009. 

As for the scoring system (computerised), its adoption in 1992 significantly reduced the rate of KOs because of the importance of the power of the punches to score, with no further changes in bouts ending before the time limit.

Also, the current use of modern materials for gloves and head guards, ringside officials, and rules guarantees higher safety for athletes and makes boxing a more spectacular sport [23,49].

Contacts to the head are a serious topic in combat sports, especially with regard to concussions. In karate, concussions are most often caused by punching and kicking techniques; in boxing, by punches; in taekwondo, by kicks; in judo, by falls on the head. In karate with no helmets, competitors are protected by the skin touch rule, compliance with which is overseen by the referee. In the age category of cadets, no hand-to-face contact is allowed during the match. Nevertheless, many points were awarded in conflict with this rule [62]. In taekwondo, a helmet is required to ensure competitors’ safety. Furthermore, in karate, face masks have recently been introduced, but their frequent damage was causing serious face lacerations. There is no regulation of mandatory helmets; therefore, there is a lack of evidence that helmets reduce injuries in karate. We think that applying the helmets during karate competitions could lead to more aggressive and even harder attacks to the head area. 

The new rules of the World Karate Federation [66] require mandatory helmets only for children in the competitions. From our point of view, it would be beneficial to research the force and frequency of head attacks in this age category. Protective helmets, alongside following the rules, can markedly prevent competitors from head injuries.

Good evidence of the effectiveness of helmets in protecting athletes from head injuries is supported by the evaluated incidence of head kicks and concussions and identified potential risk factors of concussions in sparring taekwondo [53]. The new incentive rule relating to better-awarding head kicks did not appear to increase the incidence rates of head kicks or concussions in research participants.

The rules are developed primarily to improve athlete safety, but they are also developed to enhance competition and sporting performance. However, any change of this nature needs to be carefully considered.

### 4.3. Gaps in the Existing Literature and Suggestions for Future Research on This Topic

The summary of the key areas where the current literature is lacking is provided in Table 2. In order to understand the incidence of injury, experts in combat sports injuries should have a general understanding of the rules, such as required guards, target areas, prohibited behaviour, or the duration of combat in each Olympic combat sport.

Many studies present the incidence of different injuries only as a percentage representation. This information is insufficient mainly because of different numbers of matches or, for example, the unequal duration of matches in various combat sports. 

In karate, the duration of the match is 3 min, which gives 15 min of exposure to possible injury during a tournament for an athlete if they reach the medal match. In taekwondo, the match is twice as long, specifically, 3 × 2 min, which gives 30 min of exposure to possible injury in a tournament. The longest matches take place in boxing, where they last around 52 min if the match does not end before the time limit. As a result of this discrepancy, we recommend that the authors express the incidence of injuries as a minute exposure.

It is essential to reach a balance between ensuring athlete safety and maintaining fair competition and an attractive performance. Through a comprehensive analysis of the impact of rule changes on athletic performance and injury incidence rates, it is possible to ensure that any changes implemented truly enhance the safety of the athletes and, simultaneously, the overall sporting experience. Therefore, any modifications to the rules must be thoroughly evaluated, considering potential consequences and consulting relevant stakeholders. 

Comparing epidemiological studies, we noticed methodological problems not only in the process of collecting the data but also in the selection according to the severity or anatomical location of the injuries. Some authors recognize three levels of severity (minor, mild, severe). Injuries defined as any physical complaint for which an athlete would seek assistance from tournament medical personnel were, therefore, divided by other authors as follows: non-time-loss or time-loss injury. Time-loss injuries are defined as injuries that prevent the athlete from completing the present bout and/or subsequent bouts and from participating in sports activities for a minimum of 1 day thereafter [18].

Dividing injuries between non-time-loss and time-loss injuries, in our opinion, is methodologically more sufficient. Dividing injuries into three groups according to their severity might represent a complication, especially for less experienced medical personnel. For a better comparison between studies in this field, it would be helpful to categorize injuries according to the same anatomical locations.

When authors use a uniform method of data collection, evaluation, and presentation of results, they should then consider differences in combat sports when comparing their findings. At first sight, for example, karate and taekwondo seem related sports, but the rules in these sports are completely different. Each combat sport has a different competition format, number of matches, duration, scoring areas, way of fighting, and allowed contact or target areas. For instance, in boxing or taekwondo, it is possible to win the match by KO, but in karate, contact is limited to the non-injurious; the athlete that causes a KO to their opponent would be disqualified from the competition [66]. 

It is common that the injuries are recorded and reported at the highest level of the competition or senior top-level athletes. It is important to analyze the injuries sustained at various levels of competition. Intuitively, it could be argued that athletes with a lower level of training and technique control could have more injuries. Some studies reported that higher-skilled athletes experienced more injuries, maybe because they are potentially more likely to use dangerous offensive and defensive techniques or execute fundamental ones with greater force and speed [35]. The same has been shown also in other sports, e.g., in soccer [67]. Cierna et al. [12] confirmed this, as the rate of injuries was significantly higher for U21 than for U18 categories. These differences should be monitored in epidemiological studies and also in lower levels of performance competitions, for instance, at international or regional tournaments, where athletes have lower levels of skills. This also applies to the referees and other personnel. 

Time-motion analysis involves studying the specific movements and actions performed during a sporting event. By analyzing the offensive and defensive techniques used during the injury, researchers can gain valuable insights into the potential risk factors associated with each technique. This information can also help in developing preventive measures and strategies to minimize injuries in sports. 

Furthermore, wearable sensor technology exists that can report real-time impact information. The most common types are helmet-integrated sensors, patch sensors, and mouthguard sensors, all of which measure peak linear acceleration, rotational velocity, and/or rotational acceleration [55].

As presented in Table 2, an analysis of the literature revealed several gaps in the existing studies. There is still a lack of research that seeks to investigate the relationship between protective measures and the reduction of injuries. Although the importance of injury prevention has increased over the past decade, supporting evidence is still scarce. Recently, increased research efforts have been accomplished to investigate the epidemiology of competition injuries to improve knowledge about the frequency, locations, and technical–tactical risk factors of injuries in combat sports. These researchers suggest that gender, fighter skills, tactics, or weight division could be risk factors for injuries during the combat fight, and proper protective equipment could have an impact on decreasing the occurrence of time-loss injuries in combat sports. While strict rule adherence may be a key factor in the prevention of injuries, it seems that much less evidence exists on their role in fighting competition performance and training. These gaps revealed in the literature should be addressed in future studies.

## 5. Conclusions

After the inspection of full-text articles, only seven studies were eligible for the study. A permitted head guard significantly reduces head injuries and KOs in boxing. Revoking a mandatory head guard and applying thicker gloves increases the rate of punches aimed at the head and body, along with increased movement of athletes around the ring. Rules such as reduced round duration, higher referee competencies, and computerised scoring systems reduce KO rates and injuries. In karate, the introduction of five weight divisions reduces injury incidence in the U21 category. However, the higher incentive to attack the head area does not increase the occurrence of high kicks or concussions in young taekwondo athletes. Overall it can be concluded that proper use of protective measures and rules effectively reduces injury rates in combat sports.

Most studies on injuries in combat sports focus on type, severity, location, and mechanism, with a small percentage examining the injury’s cause and how protectors or rules reduce injury likelihood. Protective measures focus on material damping capabilities, force absorption effectiveness, and testing machine validation. However, a clear relationship between injury incidence and the use of mandatory protectors and rules is lacking. Investigating the impact of protective gear and regulations on combat sports injury frequency is crucial.

The literature lacks studies on the role of rules in combat sports injury risk. Future research should focus on rules, uniform records, and protective equipment implementation. Time–motion analysis of technical and tactical causes of injuries is also needed. Studies should be conducted on lower levels of competition, different ages, genders, and technical levels. Larger sample sizes may increase power and reduce the margin of error. More elite athletes and top-level competitions should be included in future research.

## Figures and Tables

**Table 2:** Research gaps identified in the literature and suggestions for future studies.

Gaps and Limitations in the Literature	Suggestions for Future Studies
There is a lack of studies investigating the contribution of rules on the risk of injuries in combat sports.	Apart from the epidemiological studies, also investigate the contribution of rules on the risk of injuries in combat sports.
Missing uniform records form and methodology of evidence of injuries across combat sports.	To create uniform methodology and the records form for evidence of combat sports injuries.
General review of combat sports rules, duration and number of matches in the competition, target areas, scoring techniques, prohibited behaviour, protective equipment, and competition categories.	To compose a review study that will address these issues across individual Olympic combat sports.
Lack of studies conducted on the implementation of protective equipment and its contribution to the incidence rate of injuries in combat sports.	To compose the study focusing on this topic.
There is a lack of time-motion analysis of technical and tactical causes of injuries in combat sports.	To conduct a time-motion analysis of technical and tactical causes of injuries in combat sports by post-fight video analysis.
Missing studies conducted on the lower level of competition, different ages, gender, and technical levels.	To conduct studies regarding these issues.
Small sample sizes occur in most studies, which could reduce its power and increase the margin of error.	The research studies should include more elite athletes and top-level competitions.

## Data Availability

Data available in a publicly accessible repository.

## Supplementary Materials

The following supporting information can be downloaded at: https://www.mdpi.com/article/10.3390/jfmk8040150/s1.
