# Supplementary material for: Effectiveness of Protective Measures and Rules in Reducing the Incidence of Injuries in Combat Sports: A Scoping Review
Source: J Funct Morphol Kinesiol. 2023 Oct 30;8(4):150. doi: 10.3390/jfmk8040150 (PMC10660771; doi:10.3390/jfmk8040150)
Supplement: Supplementary file 1 [file jfmk-08-00150-s001.zip › jfmk-2643537-supplementary.pdf]

Table S1. Data Sources

databases	Search Terms
PubMed	Example search: Title/abstract – (combat sport OR martial arts OR Protective meatures OR rules OR protectors) AND (injuries OR injury incidence rate OR risk of injuries)<br>Limited to: Date from 2010 to 10th June 2023
Web of Science	Example search: Topic – (ALL combat sport OR ALL martial arts OR ALL Protective meatures OR ALL rules OR ALL protectors) AND (ALL injuries OR ALL injury incidence rate OR ALL risk of injuries)<br>Limited to: Date from 2010 to 10th June 2023
Scopus	Example search: Topic – (“combat sport“ OR “martial arts“ OR “Protective meatures“ OR “rules“ OR “protectors“) AND (“injuries“ OR “injury incidence rate“ OR “risk of injuries“)<br>Limited to: Date from 2010 to 10th June 2023
